# Efficient Synthesis of Fe_3_O_4_/PPy Double-Carbonized Core-Shell-like Composites for Broadband Electromagnetic Wave Absorption

**DOI:** 10.3390/polym16081160

**Published:** 2024-04-20

**Authors:** Ahmed Elhassan, Xiaoshuang Lv, Ibrahim Abdalla, Jianyong Yu, Zhaoling Li, Bin Ding

**Affiliations:** 1 State Key Laboratory for Modification of Chemical Fibers and Polymer Materials, College of Materials Science and Engineering, Donghua University, Shanghai 201620, China; salem28006@yahoo.com; 2 Shanghai Frontiers Science Center of Advanced Textiles, College of Textiles, Donghua University, Shanghai 201620, China; 3 Shanghai Key Laboratory for Development and Application of Metal Functional Materials, School of Materials Science & Engineering, Tongji University, Shanghai 201804, China; 4 Innovation Center for Textile Science and Technology, Donghua University, Shanghai 200051, China; 5 National Innovation Center of Advanced Dyeing & Finishing Technology, Tai’an 271000, China

**Keywords:** electromagnetic wave absorption, core–shell-like structure, impedance matching, Fe_3_O_4_, PPy

## Abstract

Ever-increasing electromagnetic pollution largely affects human health, sensitive electronic equipment, and even military security, but current strategies used for developing functional attenuation materials cannot be achieved in a facile and cost-effective way. Here, a unique core-shell-like composite was successfully synthesized by a simple chemical approach and a rapid microwave-assisted carbonization process. The obtained composites show exceptional electromagnetic wave absorption (EMWA) properties, including a wide effective absorption band (EAB) of 4.64 GHz and a minimum reflection loss (RL_min_) of −26 dB at 1.6 mm. The excellent performance can be attributed to the synergistic effects of conductive loss, dielectric loss, magnetic loss, and multiple reflection loss within the graphene-based core–shell-like composite. This work demonstrates a convenient, rapid, eco-friendly, and cost-effective method for synthesizing high-performance microwave absorption materials (MAMs).

## 1. Introduction

The explosion of wireless communication and ubiquitous electronics has driven electromagnetic radiation to alarming levels, posing great threats to both human health and sensitive equipment. In response, electromagnetic wave absorption (EMWA) materials have seen a significant rise in development, allowing them to capture and convert electromagnetic energy into safer forms [1,2,3]. These materials could play a vital role in addressing challenges posed by digital, satellite, and waveguide communication, apart from their military applications [4,5]. Carbon-based materials and their composites have been extensively investigated due to their exceptional EMWA characteristics. These materials are known for their high surface area, low density, and outstanding stability, which have garnered significant attention in the scientific community [6,7,8,9,10,11]. Even though various strategies have been proposed for tailoring graphene and its derivatives for EMWA applications, it still remains a big challenge to fabricate carbon-based materials using a simple, high-efficient, cost-effective, and environmentally friendly process [12].

In contrast to conventional heating methods that involve lengthy thermal treatment at high temperatures and vacuum conditions with inert gas protection, the synthesis of carbon materials through microwave irradiation has emerged as an appealing alternative. This technique offers numerous benefits, such as its eco-friendly nature, efficient heating, fast carbonization, low energy consumption, cost-effectiveness, quick start-up, and instant termination capability [13]. Microwave-assisted carbonization is based on the microwave-radiation-induced displacement of the charged particles in the material, which enables electromagnetic energy to convert into heat within the material in a contactless volumetric way. Microwave irradiation that can induce a temperature increase up to 1000 °C within even just one minute largely shortens the treatment time, which allows for time and energy conservation processes and can inhibit undesired side reactions even under heat convection [14,15]. Therefore, microwave irradiation, as a fascinating and promising tool, has opened up new avenues for green and safe carbonization by enabling innovative reaction pathways.

Polymer polypyrrole (PPy) with moderate conductivity can absorb electromagnetic energy effectively with minimal reflection, making it an ideal precursor for microwave-assisted quick carbonization to nanocarbon [16]. Herein, a core-shell-like composite with Fe_3_O_4_ and PPy as precursors was synthesized using a simple chemical approach and fast microwave calcination. The Fe_3_O_4_ within the composite acts as traps for electromagnetic waves, forming a conductive network, and serves as multi-reflected layers. Additionally, the synergistic effect between Fe_3_O_4_ and graphene optimizes the impedance matching. This core–shell-like composite demonstrates exceptional EMWA performance, with an RL_min_ of −26 dB at 16 GHz at 1.6 mm and an extensive EAB of 4.64 GHz, which can be attributed to the synergistic effect of conductive loss, dielectric loss, magnetic loss, and multiple reflection loss. This study presents a simple and effective strategy for producing core–shell-like composites with outstanding EMWA capability.

## 2. Materials and Experimental Methods

### 2.1. Materials

Pyrrole monomer (99%), iron(III) chloride (FeCl_3_, 98%), triethylene glycol (99%), iron (III) acetylacetonate, and polyvinylpyrrolidone (PVP, Mw 1,300,000) were all supplied by Shanghai Aladdin Chemical Co., Ltd., Shanghai, China.

### 2.2. Preparation of the Carbonized PPy Nanospheres (CNs)

The preparation of CNs included the synthesis of PPy nanospheres (PNs) followed by the carbonization process. Specifically, 1 g of pyrrole monomer was homogeneously dispersed in 40 mL of deionized water by magnetic stirring. After the addition of a mixture of 20 mL of FeCl_3_ and deionized water (in a ratio of 1:20), the pyrrole polymerization process was initiated. The polymerization lasted for 3 h, with the solution being mechanically stirred in an ice-water bath at approximately 5 °C. The as-synthesized dark precipitate of PN was collected by suction, washed with distilled water and ethanol several times, and finally dried at 60 °C for 12 h. In contrast to traditional carbonization approaches, a rapid calcination process was proposed by using a domestic microwave oven with an 800 W output power. A total of 150 mg of PN was put in the microwave oven with continuous irradiation for 15 s. After approximately 10 s, electric arcing occurred along the PN, resulting in a rapid increase in temperature. Sparks generated during such arcing can reach exceptionally high temperatures, often exceeding 1000 °C [17,18]. This intense heat effectively carbonized the PN, leading to the formation of CNs.

### 2.3. Preparation of the Functionalized CN (FC)

To fabricate FC, 150 mg of CNs was firstly dispersed in 40 mL of triethylene glycol solution under ultrasonic for 30 min to obtain a uniform suspension. A total of 1 g of iron (III) acetylacetonate Fe(acac)_3_ was dissolved in 20 mL of triethylene glycol, which was then introduced into the CN suspension and ultrasonicated for over 1 h. Subsequently, the resulting solution was heated to 270 °C with vigorous stirring under argon protection. The resulting FC was obtained through magnetic separation, washed multiple times with ethanol, and dried in a vacuum oven at 80 °C for 12 h.

### 2.4. Preparation of Double-Carbonized FC (CFC)

The in situ chemical polymerization and microwave carbonization were employed to prepare the PPy coated FC (PFC). Briefly, 150 mg of freshly prepared FCs was dispersed in a 0.2 wt% solution of PVP and subjected to ultrasonication at room temperature for 1 h. Subsequently, 1 g of pyrrole monomer was added to the FC suspension and ultrasonicated for another 30 min. A mixture containing 0.5 g of FeCl_3_ and 5 mL of deionized water was slowly added with ultrasonication for 30 min. The resulting PFC precipitates coated with PVP and PPy were filtered, washed with ethanol and distilled water, and ultimately dried at 80 °C for 12 h. Finally, 50 mg of as-prepared material was subjected to a second microwave irradiation process. Continuous irradiation for 1 min resulted in the successful production of black CFC powder.

### 2.5. Characterization and Measurements

The core–shell composites were extensively analyzed using various techniques, including scanning electron microscopy (SEM), high-resolution transmission electron microscopy (TEM), X-ray diffraction (XRD), Fourier transform infrared spectroscopy (FT-IR), Raman spectroscopy, thermogravimetric analysis (TGA), and vector network analysis. The relative permittivity and permeability of the composites were determined using a vector network analyzer in the frequency range of 2–18 GHz. Additional details about the instruments employed and the sample preparation can be found in Text S2 (see Supplementary Materials).

## 3. Results and Discussion

**Figure 1:**
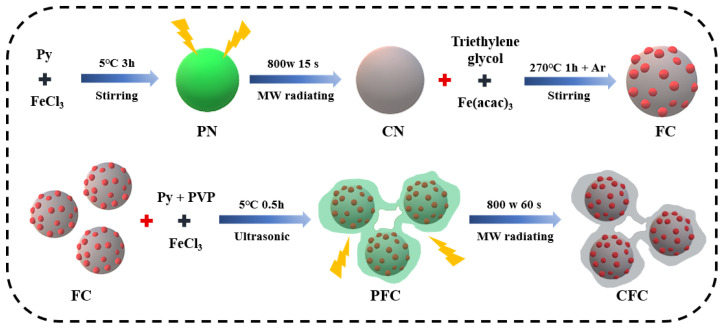
Schematic illustration of preparation process for the core-shell-like structure composites.

The composite materials had a core-shell-like structure, consisting primarily of multi-shell components using conductive PPy as the precursor for carbon. As illustrated in Figure 1, this distinctive hybrid structure was successfully fabricated simply and rapidly. Initially, the PN was synthesized through chemical polymerization. Subsequently, the PN was carbonized in a household microwave oven for 15 s under ambient conditions, resulting in the formation of a CN. To further enhance its properties, a dense magnetic layer composed of Fe_3_O_4_ nanoparticles (NPs) was introduced to the CN through a decomposition process, leading to the creation of an FC. Following that, a layer of PPy was coated onto the FC, forming a PFC. Finally, the PFC underwent carbonization in a household microwave oven for approximately 60 s, resulting in the production of a CFC.

### 3.1. Morphology and Structure Analysis of Core–Shell-like Composites

**Figure 2:**
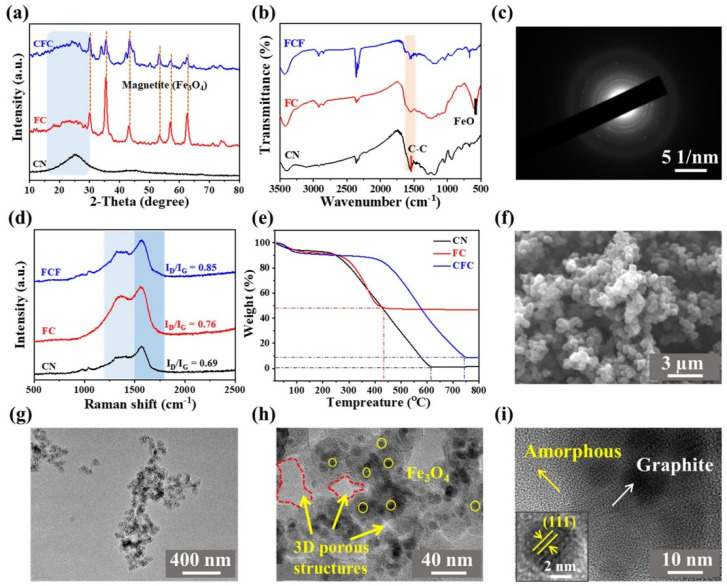
(**a**) XRD spectra, (**b**) FT-IR spectra, (**c**) SAED pattern, (**d**) Raman spectra, and (**e**) TGA curves of core-shell-like structured composites. (**f**) SEM image and (**g**–**i**) TEM images of core–shell-like structured composites.

Figure 2 provides a comprehensive analysis of the composition, thermal properties, morphologies, and structures of the core-shell-like composite. In Figure 2a, X-ray diffraction (XRD) reveals broad peaks at 26° and 44°, which are characteristic of carbon materials [19]. This observation is further supported by the FTIR analysis shown in Figure 2b, where a characteristic peak at 1560 cm^−1^ is consistent with carbonized PNs [20]. The presence of small Fe_3_O_4_ NPs (approximately 12 nm) on the FC is confirmed by the characteristic XRD marked peaks and the FTIR peak at 585 cm^−1^, corresponding to the FeO bond. This functionalization is visualized in Figure S2 (see Supplementary Materials) [21]. Furthermore, Figure 2c displays the selected area electron diffraction (SAED) pattern, which exhibits distinct diffraction ring patterns. This indicates the formation of Fe_3_O_4_ nanocrystals with a body-centered cubic (bcc) phase, randomly distributed as a result of carbonization [22]. The response of the samples to a magnet, as shown in Figure S1 (see Supplementary Materials), provides compelling evidence for the presence of Fe_3_O_4_ NPs. In Figure 2d, the Raman spectra show an increased intensity ratio of the D band to the G band (D/G ratio) for the FC and CFC. This suggests that the presence of Fe_3_O_4_ NPs leads to a catalytic graphitization effect, promoting the formation of graphitic carbon structures in the samples [23,24]. As can be seen in Figure 2e, the thermal stability of FC and CFC samples increased significantly compared with CNs due to rapid double-carbonization and the functionalized Fe_3_O_4_ layer on the CN surface. These findings suggest that the synthesized materials have good thermal stability and potential for various applications.

The confirmation of morphologies and microstructures of the core-shell-like composites can be achieved through the utilization of SEM and high-resolution transmission electron microscopy (HR-TEM). As depicted in Figure 2f–i, the composite comprises carbon nanospheres measuring approximately 260 nm in diameter with Fe NPs uniformly distributed within the structure. The interlocked 3D porous structures with a few nanometer lengths are also observed among the chain-like branched structure (Figure 2g). The HR-TEM image exhibits ordered graphite layers surrounded by metal NPs with a lattice of single-crystalline Fe_3_O_4_ (Figure 2i) [25]. The amorphous region refers to the disordered arrangement of carbon atoms within the layers of graphite, specifically related to the turbostratic nature of graphitic planes.

### 3.2. EMWA Performance of the Core–Shell-like Composites

**Figure 3:**
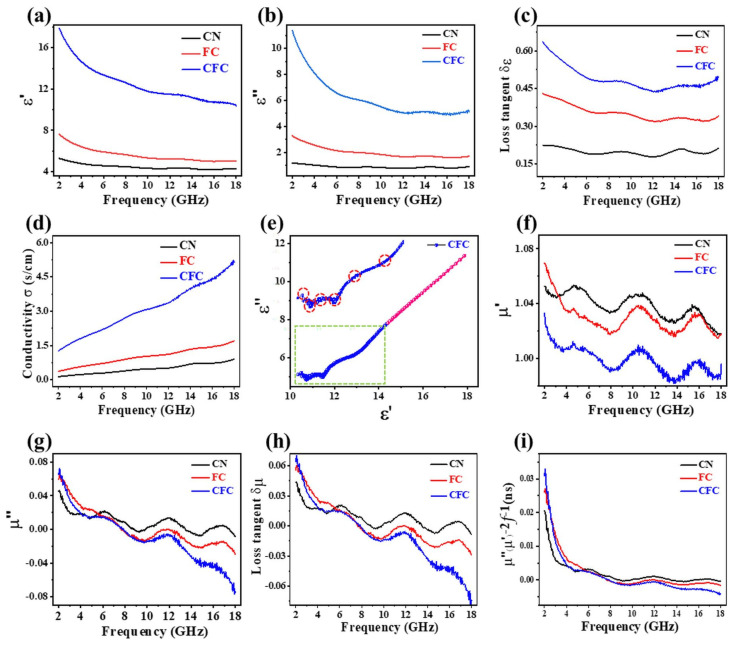
Frequency dependence of (**a**,**b**) the complex permittivity and (**c**) dielectric loss tangent, and (**d**) conductivity. (**e**) The ε′–ε″ curve of CFC. (**f**,**g**) The complex permeability, (**h**) magnetic loss tangent, and (**i**) (μ″(μ′) − 2f − 1) curve.

The assessment of electromagnetic wave absorption (EMWA) properties is greatly dependent on the values of complex permittivity and permeability. These crucial parameters can be determined by utilizing the equations provided in Equations (S3) and (S4) of the Supplementary Materials file. Furthermore, for a comprehensive analysis and validation of electromagnetic wave absorption performance, we have included all the relevant equations in Text S3 of the Supplementary Materials file. In this study, we examined three samples depicted in Figure 3a,b. Our observations reveal that the complex permittivity exhibits a slight decrease as the frequency increases. This finding suggests that the molecular response may not effectively adapt to rapidly changing fields at higher frequencies [26,27,28]. In Figure 3b, the CFC sample demonstrates the highest dissipation value compared to FC and CN samples, which can be ascribed to the presence of Fe_3_O_4_ within the carbon layers, thus resulting in ideal impedance matching. The presence of Fe_3_O_4_, along with abundant defects and non-graphitic carbon, significantly enhances dipole polarization, creates multiple polarized centers, and introduces numerous interfaces between Fe_3_O_4_ and graphene, as well as amorphous defect graphite regions [29,30]. These interfaces induce interfacial polarization effects, leading to charge redistribution, dipole formation, and energy dissipation through molecular or interfacial relaxations. In addition, as shown in Figure 3c,d, the introduction of the core-shell-like structure with embedded Fe_3_O_4_ NPs improves the conductivity and provides a network model to transport the aggregation-induced charge, which significantly contributes to the dielectric loss performances [31]. The Cole-Cole plots (Figure 3e and Figure S3 (see Supplementary Materials)) unveil the CFC’s greater dielectric loss accessibility that is manifested by the extensive semicircle overlaps, while slender tails indicate the interplay of dominant conductive loss. Therefore, the conductive loss and the dielectric loss corporately promote the final EMWA performance [32,33].

The complex permeability of the three samples demonstrates a consistent decreasing trend accompanied by resonance peaks (Figure 3f,g). Notably, the CN sample exhibited values close to zero for both permeability and tangent loss across all frequencies, whereas the FC and CFC displayed higher values at low frequencies that decreased significantly and became negative as the frequency increased (as seen in Figure 3g,h). The substantial drop in μ″ and tan δμ can be attributed to the unique microstructure and composition of the FC and CFC. These samples possess a distinctive architecture, including a complex core-shell structure with various-sized pores and disordered graphite layers, along with the presence of FeO_x_ NPs. During testing, the complex structure scatters the incident wave in different directions, resulting in interference effects. This scattering and interference generate an induced magnetic field that opposes the applied field, leading to a negative permeability. In addition to the scattering-induced negative permeability, there is also a contribution from magneto-electric coupling. This coupling arises from the interaction between magnetic and electric dipoles within the material [2,34,35,36,37]. The fluctuation behavior observed in Figure 3i is attributed to a combination of natural resonance and eddy current loss [38]. The magnetic loss within the 2–5 GHz range is attributed to natural resonance, while the consistent magnetic loss observed above 8 GHz is linked to eddy current loss. At higher frequencies, the hybrid nanosphere’s eddy current loss becomes the dominant source of magnetic loss by dissipating incident microwave energy [39,40]. Consequently, Figure 3c,h demonstrate that dielectric loss plays a dominant role in EMWA, surpassing magnetic loss (tan δε > tan δμ) consistently across all frequencies.

**Figure 4:**
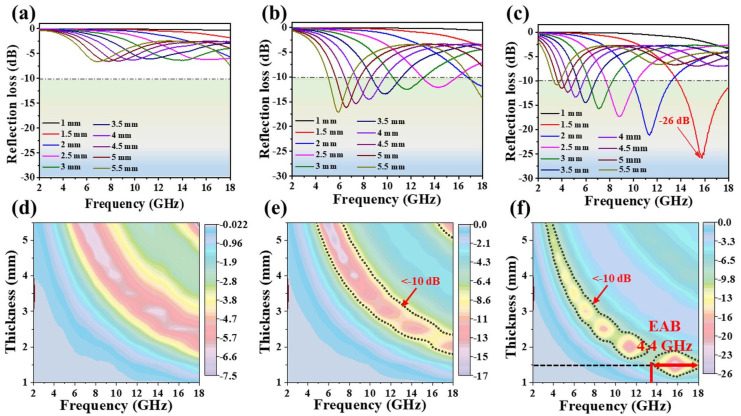
RL curves of CN (**a**,**d**), FC (**b**,**e**), and CFC (**c**,**f**) at different matching thickness.

**Figure 5:**
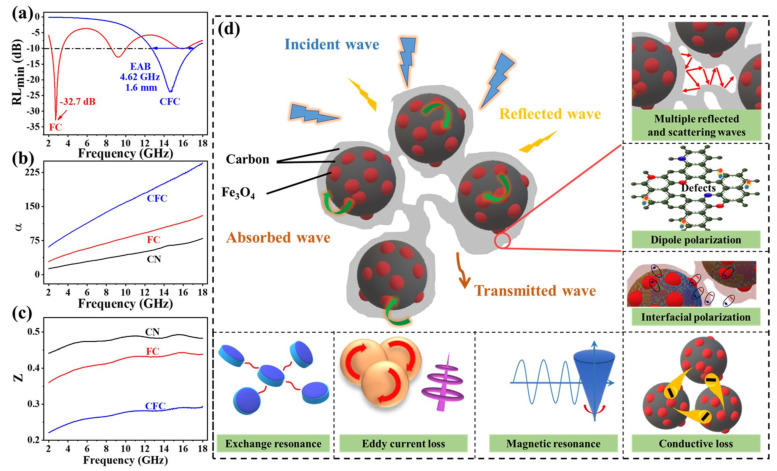
Frequency dependence of (**a**) contrast diagram of the location of RL_min_ and EAB values, (**b**) attenuation constant, and (**c**) impedance matching ratio. (**d**) Schematic illustration for the microwave absorbing mechanisms.

Figure 4 showcases the performance of three different samples (CN, FC, and CFC) across the frequency range of 2–18 GHz with varying thicknesses ranging from 1 to 5 mm. Across various thicknesses, the FC and CFC samples demonstrate a remarkable EMWA performance, achieving an RL value below −10 dB and highlighting their exceptional absorption efficiency (Figure 4b,c,e,f). In contrast, the CN sample exhibits comparatively lower microwave absorption capabilities, as illustrated in Figure 4a,d. Particularly, the CFC sample demonstrates the highest absorption capability, achieving an EAB of 4.4 GHz and RL_min_ of −26 dB at 16 GHz with a thin thickness of only 1.5 mm (Figure 4c,f). On the other hand, the FC sample achieves an RL_min_ of −32.7 dB (99.99% dissipation efficiency) at a lower frequency of 2.7 GHz, but it requires a thickness exceeding 10 mm (see Figure S4 in the Supplementary Materials). By comparing Figure 4b,c with Figure S4, it becomes evident that the CFC sample exhibits an EAB of 14.8 GHz (3.2–18 GHz) within the thickness range of 1.5 to 5.5 mm. In contrast, the FC sample demonstrates an EAB of 15.8 GHz (2.2–18 GHz), but within a relatively higher thickness range of 1.5 to 10 mm. Furthermore, the CFC sample displays a maximum EAB of 4.62 GHz, corresponding to an RL_min_ of −24 dB, at a thickness of only 1.6 mm (Figure 5a). The unique core-shell-like structures, along with various-sized pores and disorder in turbostratic graphite, contribute to a broadened absorption bandwidth, making the composite suitable for broadband EMWA applications. In general, along with their wide EAB and thin thickness advantage, these exceptionally rapid double-carbonized core-shell-like samples offer several advantages compared to earlier findings in the field of EMWA materials (for instance, in [9]), which were prepared using more intricate, energy-intensive, and time-consuming methods.

Therefore, the attenuation and impedance results shown in Figure 5b,c) align with the observed absorption performance of the CFC. Notably, the CFC demonstrates higher attenuation and lower impedance compared to the other samples, enabling a larger portion of incident waves to be absorbed and simultaneously converted into heat. As a result, the reflection and transmission are reduced, leading to an enhanced absorption efficiency. However, the combination of core-shell-like structures, 3D porosity, carbon defects, and the embedded Fe_3_O_4_ NPs results in synergistic electromagnetic loss mechanisms, as illustrated in Figure 5d. These mechanisms collectively enhance the EMWA performance, broaden the absorption range, and facilitate efficient energy dissipation by optimizing the interaction and minimizing reflection and transmission waves.

## 4. Conclusions

In summary, by subjecting PPy to double-microwave-radiation, a core–shell-like structure consisting of an inner magnetic shell layer was successfully fabricated within a short period of time. The unique combination of magnetic and dielectric materials in the hybrid composites enables exceptional electromagnetic wave absorption thanks to diverse interactions within the structures. Comparing the CN and FC samples, which exhibited a minimum reflection loss of −6.7 dB and −16.9 dB, respectively, despite their relatively large thickness of 5.5 mm, the CFC sample demonstrated remarkable performance. It achieved an impressive minimum reflection loss of −26 dB at 16 GHz with an effective absorption band of 4.64 GHz at only 1.6 mm, covering a broad absorption range of 2.5–18 GHz. In contrast to EMWA materials fabricated using conventional carbonizing methods, the ultrafast double-carbonized core-shell-like samples demonstrate remarkable efficiency. Their eco-friendly nature, efficient heating, rapid carbonization, and low energy consumption contribute to their superior performance. This work unveils a scalable and cost-effective approach to designing advanced EMWA materials, offering an efficient alternative with sophisticated structures.

## Data Availability

Data are contained within the article and Supplementary Materials.

## Supplementary Materials

The following supporting information can be downloaded at: https://www.mdpi.com/article/10.3390/polym16081160/s1, Figure S1: Magnetic attraction of (a) CN, (b) FC, and (c) CFC; Figure S2: Fe_3_O_4_ NPs diameter; Figure S3: The Cole-Cole curve of (a) CN and (b) FC samples; Figure S4: RL curves of (a) FC and (b) CFC samples; Text S1: Carbon-based materials used in EMWA application; Text S2: Characterization and Measurements; Text S3: Electromagnetic analysis; XRD analysis: Equation (S1); Equation for Calculating Electric Conductivity (σ): Equation (S2); Equations for Analyzing and Validating Electromagnetic Wave Absorption Performance: Equations (S3)–(S10); References [41,42,43,44,45,46,47,48] are cited in the supplementary materials.
