# Supplementary material for: Efficient Synthesis of Fe3O4/PPy Double-Carbonized Core-Shell-like Composites for Broadband Electromagnetic Wave Absorption
Source: Polymers (Basel). 2024 Apr 20;16(8):1160. doi: 10.3390/polym16081160 (PMC11053598; doi:10.3390/polym16081160)
Supplement: Supplementary file 1 [file polymers-16-01160-s001.zip › polymers-2957037-supplementary.pdf]

Supplementary Materials

Efficient Synthesis of Fe_3O_4/PPy Double-Carbonized Core–Shell-Like Composites for Broadband Electromagnetic Wave Absorption

Ahmed Elhassan [1], Xiaoshuang Lv [2], Ibrahim Abdalla [4], Jianyong Yu [5], Zhaoling Li [2,3*], Bin Ding [5*]

[1] State Key Laboratory for Modification of Chemical Fibers and Polymer Materials, College of Materials Science and Engineering, Donghua University, Shanghai 201620, China.

[2] Shanghai Frontiers Science Center of Advanced Textiles, College of Textiles, Donghua University, Shanghai 201620, China.

[3] National Innovation Center of Advanced Dyeing & Finishing Technology, Tai'an, Shandong 271000, China

[4] Shanghai Key Laboratory for Development and Application of Metal Functional Materials, School of Materials Science & Engineering, Tongji University, Shanghai, 201804, China

[5] Innovation Center for Textile Science and Technology, Donghua University, Shanghai 200051, China.

* Corresponding authors. E-mail: zli@dhu.edu.cn (Z. Li); binding@dhu.edu.cn (B. Ding)

Text S1: Carbon-based materials used in EMWA application

Various carbon nanomaterials exhibit excellent characteristics for electromagnetic wave absorption (EMWA) due to their high surface area, low density, and high stability. Fabricating unique nanocarbon materials usually requires pyrolysis of carbon precursors like aromatic polyimide film and poly(acrylonitrile), but current methods are expensive and challenging to scale up due to harsh conditions and complex equipment [1-3]. Recently, microwave irradiation methods have been proposed as an alternative to conventional heating approaches for fabricating carbon nanostructures. Domestic microwave oven calcination offers several advantages, such as rapid and volumetric heating, economical and eco-friendly reactions, and the promotion of advanced material fabrication. The synthesis of carbon materials via microwave irradiation has recently become a promising alternative due to its green feature enabled by high-efficiency heating, fast carbonization, low energy consumption, low processing cost, quick start-up and stopping, etc. Microwave-assisted carbonization is based on the microwave-radiation-induced displacement of the charged particles in the material, which enables electromagnetic energy to convert into heat within the material in a contactless volumetric way. Microwave irradiation that can induce a temperature increase up to 1000°C within even just one minute largely shortens the treatment time, which allows for time and energy conservation processes and can inhibit undesired side reactions even under heat convection. Therefore, microwave irradiation, as a fascinating and promising tool, has enabled a new reaction pathway for green and safe carbonization [4,5]. However, PPy's moderate conductivity plays a crucial role in the rapid and efficient microwave-assisted conversion to nanocarbons, acting as a

bridge between the incident microwave energy and the carbon precursor. Ppy's conjugated π-electron system allows for efficient energy absorption, enabling it to readily interact with the electric field of the microwave radiation and convert the electromagnetic energy into heat through dipole polarization within its structure. This moderate conductivity prevents excessive reflection of the microwaves, ensuring deeper penetration and more efficient utilization of the energy within the precursor material.

In addition, despite their low cost and strong magnetic properties, magnetic materials like Fe_3O_4 have drawbacks that limit their practical applications. Incorporating magnetic nanoparticles into nanocarbon materials is expected not only to overcome these limitations but also to improve the overall properties of the nanocomposites, making them attractive candidates for efficient electromagnetic wave absorption [6].

Text S2: Characterization and Measurements

Surface morphologies and structures of the as-prepared core–shell-like composites were investigated with a scanning electron microscope (Hitachi S-4800, Japan) and a high-resolution transmission electron microscope (JEOL JEM-2100, Japan), respectively. The crystal structure analysis of the composites was characterized using X-ray diffraction (XRD) (Dandong Tongda D-3500, China). Fourier transform infrared (FT-IR) spectroscopy (JASCO, FT-IR 4200, USA) was performed to characterize the chemical structure. Raman spectroscopy (Renishaw Via-Reflex, UK) was performed. The thermal decomposition was investigated by the thermogravimetric analysis (TGA) (DSC SDT Q600, USA) with a heating rate of 10 °C/min from 25 °C to 800 °C under air atmosphere. A vector network analyzer (Agilent PNA-N5244A, USA) was applied to determine the relative permittivity and permeability in the frequency range of 2-18 GHz for the calculation of the reflection loss (RL). To ensure good quarter-wavelength matching, the measured samples were prepared by pressing homogeneous mixtures of 90 wt% paraffin and 10 wt% sample powders into cylindrical specimens with precise dimensions (3.04 mm inner diameter, 7 mm outer diameter, and 2 mm thickness).

XRD analysis

Scherrer's formula is an important tool in X-ray crystallography for determining the average size of nanocrystalline particles or crystallites. It analyzes the broadening of X-ray diffraction peaks to calculate particle size. In the specific case of Fe_3O_4 nanoparticles (NPs), Scherrer's formula was used to estimate their size, resulting in a calculated size of approximately 8 nm. Scherrer's formula is as follows:

$$L = k\lambda/(\beta\cos\theta) \qquad \text{(S1)}$$

where L represents the average crystallite size or particle size, and k is the Scherrer constant, typically ranging from 0.9 to 1.0. It takes into account various factors, such as the shape of the crystallites and instrumental broadening. λ is the wavelength of the X-ray radiation used for the analysis. β is the full-width at half-maximum (FWHM) of the diffraction peak, which is a measure of the peak broadening, and θ is the Bragg angle, defined as the angle between the incident X-ray beam and the reflecting planes in the crystal [7].

Text S3: Electromagnetic analysis

Equation for Calculating Electric Conductivity (σ):

$$\sigma = 2\pi f \varepsilon_r'' \varepsilon_0 \quad \text{(S2)}$$

where σ is the electric conductivity of the material, f referes to the frequency of the microwave radiation, and ε_r'' and ε_0 represent the imaginary part of the complex relative permittivity and the vacuum permittivity, respectively. This equation relates the conductivity to the imaginary part of the complex relative permittivity and the frequency of the microwave radiation. It is commonly used to estimate the conductivity of materials based on their dielectric properties and the operating frequency of the microwave [8,9].

Equations for Analyzing and Validating Electromagnetic Wave Absorption Performance:

The relative complex permittivity *(ε_r)* is as follows:

$$\varepsilon_r = \varepsilon' - j\varepsilon'' \quad \text{(S3)}$$

The relative complex permeability (μ_r) is as follows:

$$\mu_r = \mu' - j\mu'' \quad \text{(S4)}$$

The dielectric loss tangent ($tan\delta_\varepsilon$) and magnetic loss tangent ($tan\delta_\mu$) are used to characterize the EM loss.

The dielectric loss tangent is as follows:

$$tan\,\delta_\varepsilon = \varepsilon''/\varepsilon' \quad \text{(S5)}$$

The magnetic loss tangent is as follows:

$$tan(\delta_\mu) = \mu''/\mu' \quad \text{(S6)}$$

Debye relaxation theory is as follows:

$$\left(\varepsilon' - \frac{\varepsilon_s+\varepsilon_\infty}{2}\right)^2 + \varepsilon''^2 = \left(\frac{\varepsilon_s-\varepsilon_\infty}{2}\right)^2 \quad \text{(S7)}$$

The absorption ability of electromagnetic waves (EMWs) is often quantified using the reflection loss (*RL*) factor.

$$RL(d) = 20lg\left|\frac{Z_{in}-Z_0}{Z_{in}+Z_0}\right| \quad \text{(S8)}$$

The input impedance is as follows:

$$Z_{in} = Z_0\sqrt{\frac{\mu_r}{\varepsilon_r}}tanh\left(j\frac{2\pi fd}{c}\sqrt{\mu_r\varepsilon_r}\right) \quad \text{(S9)}$$

The equation for the attenuation constant (α) can be expressed as follows:

$$\alpha = \frac{\sqrt{2}}{c}\pi f\sqrt{(\varepsilon''\mu'' - \varepsilon'\mu') + \sqrt{(\varepsilon''\mu'' - \varepsilon'\mu')^2 + (\varepsilon''\mu'' + \varepsilon'\mu')^2}} \quad \text{(S10)}$$

wherein ε' and ε'' represent the real part of the permittivity and the imaginary part of the permittivity, respectively, μ' and μ'' are the real part of permeability and the imaginary part of permeability, respectively, σ is conductivity, d represents the thickness of the absorber, Z_0 is the impedance of air (free space) or the output impedance of the absorber, c is the velocity of light, α represents the attenuation constant, and ε_s and ε_∞ refer to the static dielectric constant and the dielectric constant at infinite frequency, respectively.

The -10 dB and -20 dB reflection loss represent the 90 and 99% electromagnetic waves absorbed by the materials, respectively [10,11].

Figures and Captions

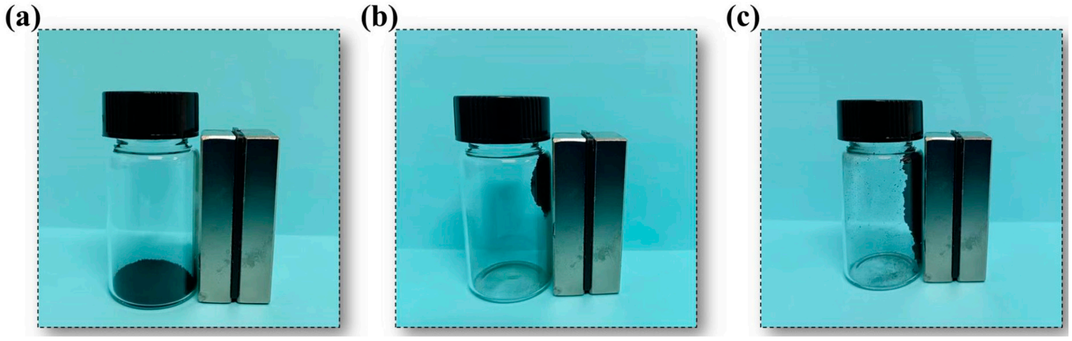

Figure S1. Magnetic attraction of (a) CN, (b) FC, and (c) CFC.

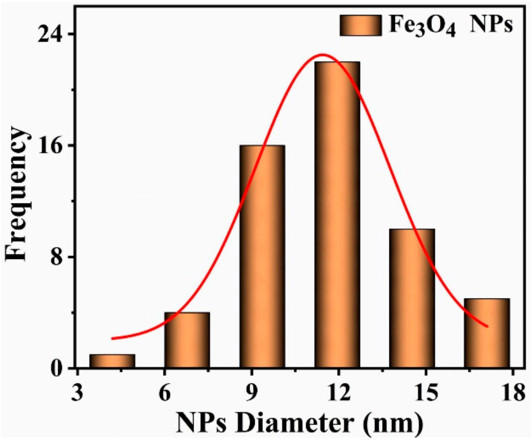

Figure S2. Fe_3O_4 NPs diameter.

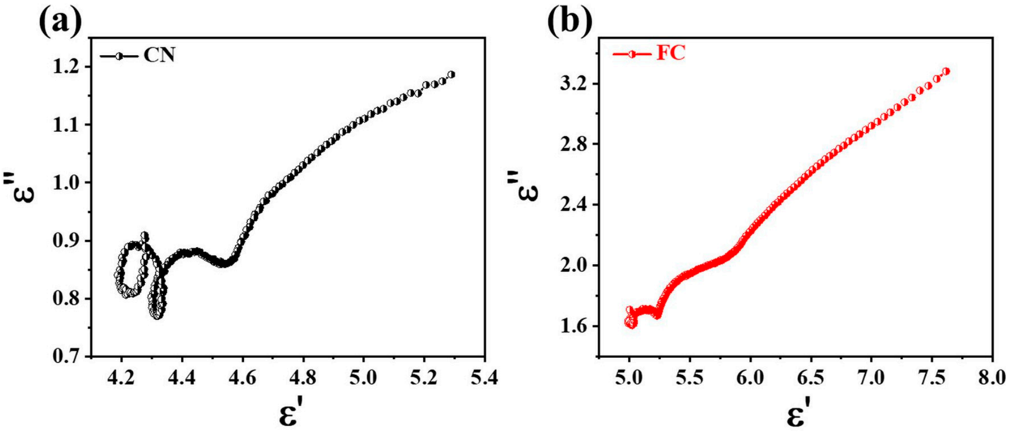

Figure S3. The Cole–Cole curve of (a) CN and (b) FC samples.

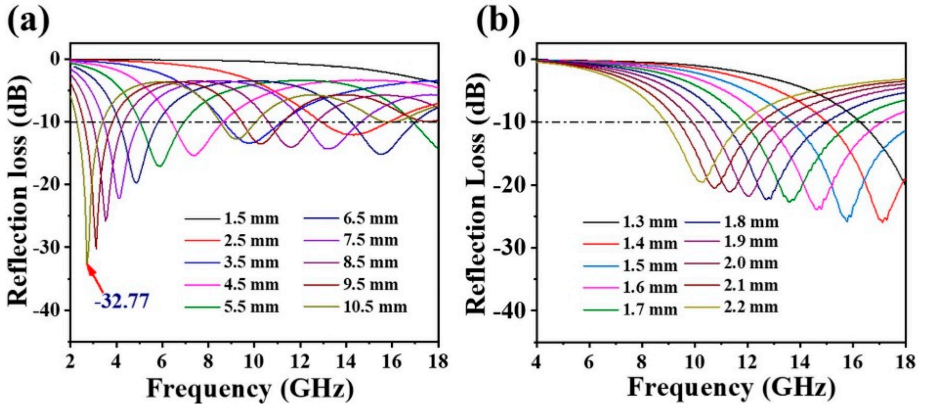

Figure S4. RL curves of (a) FC and (b) CFC samples.

References

1. Zheng, W.; Zhang, P.; Chen, J.; Tian, W.B.; Zhang, Y.M.; Sun, Z.M. In situ synthesis of CNTs@Ti_3C_2 hybrid structures by microwave irradiation for high-performance anodes in lithium ion batteries. *J. Mater. Chem. A* **2018**, *6*, 3543-3551, doi:10.1039/C7TA10394H.
2. Nie, H.; Cui, M.; Russell, T.P. A route to rapid carbon nanotube growth. *Chem. Commun.* **2013**, *49*, 5159-5161, doi:10.1039/C3CC41746H.
3. Raza, A.; Wang, J.; Yang, S.; Si, Y.; Ding, B. Hierarchical porous carbon nanofibers via electrospinning. *Carbon Lett.* **2014**, 15, 1-14, https://doi.org/10.5714/CL.2014.15.1.001.
4. Rezvanpanah, E.; Ghaffarian Anbaran, S.R.; Di Maio, E. Carbon nanotubes in microwave foaming of thermoplastics. *Carbon* **2017**, *125*, 32-38, doi:https://doi.org/10.1016/j.carbon.2017.09.035.

5. Zhu, Y.-J.; Chen, F. Microwave-assisted preparation of inorganic nanostructures in liquid phase. *Chem. Rev.* **2014**, *114*, 6462-6555, doi:10.1021/cr400366s.
6. Zhu, J.; Pallavkar, S.; Chen, M.; Yerra, N.; Luo, Z.; Colorado, H.A.; Lin, H.; Haldolaarachchige, N.; Khasanov, A.; Ho, T.C.; et al. Magnetic carbon nanostructures: microwave energy-assisted pyrolysis vs. conventional pyrolysis. *Chem. Commun.* **2013**, *49*, 258-260, doi:10.1039/C2CC36810B.
7. Abdalla, I.; Salim, A.; Zhu, M.; Yu, J.; Li, Z.; Ding, B. Light and flexible composite nanofibrous membranes for high-efficiency electromagnetic absorption in a broad frequency. *ACS Appl. Mater. Interfaces* **2018**, *10*, 44561-44569, doi:10.1021/acsami.8b17514.
8. Elhassan, A.; Abdalla, I.; Yu, J.; Li, Z.; Ding, B. Microwave-assisted fabrication of sea cucumber-like hollow structured composite for high-performance electromagnetic wave absorption. *Chem. Eng. J.* **2020**, *392*, 123646.
9. Mordina, B.; Kumar, R.; Tiwari, R.K.; Setua, D.K.; Sharma, A. Fe_3O_4 nanoparticles embedded hollow mesoporous carbon nanofibers and polydimethylsiloxane-based nanocomposites as efficient microwave absorber. *J. Phys. Chem. C* **2017**, *121*, 7810-7820, doi:10.1021/acs.jpcc.6b12941.
10. Abdalla, I.; Cai, J.; Lu, W.; Yu, J.; Li, Z.; Ding, B. Recent progress on electromagnetic wave absorption materials enabled by electrospun carbon nanofibers. *Carbon* **2023**, *213*, 118300, doi:https://doi.org/10.1016/j.carbon.2023.118300.
11. Li, T.; Zhi, D.; Chen, Y.; Li, B.; Zhou, Z.; Meng, F. Multiaxial electrospun generation of hollow graphene aerogel spheres for broadband high-performance microwave absorption. *Nano Res.* **2020**, *13*, 477-484, doi:10.1007/s12274-020-2632-0.
